# Mechanistic Studies of CO_2_ Cycloaddition Reaction Catalyzed by Amine-Functionalized Ionic Liquids

**DOI:** 10.3389/fchem.2019.00615

**Published:** 2019-09-10

**Authors:** Jian Chen, Han Gao, Tong Ding, Liangzheng Ji, John Z. H. Zhang, Guohua Gao, Fei Xia

**Affiliations:** ^1^Shanghai Key Laboratory of Green Chemistry and Chemical Processes, Shanghai Engineering Research Center of Molecular Therapeutics and New Drug Development, School of Chemistry and Molecular Engineering, East China Normal University, Shanghai, China; ^2^NYU-ECNU Center for Computational Chemistry at NYU Shanghai, Shanghai, China

**Keywords:** CO_2_ conversion, amine-functionalized ionic liquids, cyclic carbonates, DFT calculations, mechanism

## Abstract

The homogeneous cycloaddition reaction of CO_2_ and epichlorohydrin catalyzed by amine-functionalized ionic liquid (AFIL) to yield cyclic carbonate is reported in this study. The AFIL has the dual function of ionic liquid and organic base. The experimental study indicates that AFIL can efficiently catalyze the conversion of CO_2_ and epichlorohydrin to the product 3-chloro-1,2-propylene. The mechanistic study based on DFT calculations reveals that the imidazolium ring in AFIL primarily catalyzes the ring-opening reaction of epichlorohydrin, while the protonated amine group is responsible for stabilizing the Br^−^ anion in the nucleophilic attack. This study provides a deep insight into the catalytic roles of AFIL and also inspires us to design efficient dual function catalysts for CO_2_ utilization.

## Introduction

The concentration of carbon dioxide (CO_2_) in the atmosphere continues to increase due to the deforestation and burning of fossil fuels, which makes earth's environment worse than before (Hunt et al., 2010; Monastersky, 2013). The rational conversion of CO_2_ to valuable chemicals has attracted wide attentions all over the world in decades (Rochelle, 2009; Jones, 2011). Since the carbon-oxygen bonds in CO_2_ are quite stable in thermodynamics, CO_2_ is too inert to be activated (Aresta et al., 2013; Chong and Kinjo, 2015). One of effective methodologies for CO_2_ conversion is to use high-energy materials such as epoxides to react with CO_2_ to yield oxidized low-energy synthetic targets (Sakakura et al., 2007). The epichlorohydrin, as one of significant functional epoxides (Padwa and Murphree, 2005; D′Elia et al., 2015), is the main raw material for the synthesis of epoxy resin and glycerol in chemical industry. Thus, it is desirable to perform the cycloaddition of CO_2_ with epoxides to give rise to the valuable chemicals such as cyclic carbonates.

Various homogeneous catalysts including metal salts (Ma et al., 2012), organic bases (Yano et al., 1997), and ionic liquids (Sheldon, 2001; Song et al., 2014) have been applied to promote the cycloaddition reactions of CO_2_ with epoxides. Among these catalysts, ionic liquids have a profound effect on activity and selectivity. Ionic liquid catalysts are deeded as considerable potential materiel in industrial application. Ionic liquids are effective solvents or catalysts for the cycloaddition of CO_2_ with epoxides on account of its unique characters such as thermal and chemical stability, selective solubility toward organic and inorganic materials, and high recyclable.

Peng reported the reaction of quantitative conversion of propylene oxide (PO) to propylene carbonates (PC) catalyzed by 1-butyl-3-methyl-imidazolium (BMimBF_4_) ionic liquid (Peng and Deng, 2001). Xiao achieved the chemical conversion of CO_2_ with epoxides to yield cyclic carbonates under mild conditions by using immobilized ionic liquid catalyst (Xiao et al., 2006). Sun synthesized a series of hydroxyl-functionalized ionic liquids which possess efficient reactivity toward the coupling of epoxide and CO_2_ (Sun et al., 2008). Wang reported the one-pot conversion of CO_2_, ethylene oxide (EO) and amines to 3-aryl-2-oxazolidinones, which were catalyzed by the binary ionic liquids composed of 1-butyl-3-methyl-imidazolium bromide (BmimBr) and 1-butyl-3-methyl-imidazolium acetate (Wang et al., 2013). Appayuri groups developed a facile and efficient synthesis of styrene carbonate via cycloaddition of CO_2_ to styrene oxide over ordered mesoporous MCM-41-Imi/Br catalyst (Appaturi and Adam, 2013). Anthofer et al. (2014, 2015) synthesized hydroxy-functionalized mono- and bis-imidazolium bromides for the cycloaddition traction between CO_2_ and epoxides and found that the activity of the latter was higher than that of the former. Elageed et al. (2016) developed a selective procedure for the synthesis of 5-substituted N-aryloxazolidinones by mixing the epoxides with arylcarbamates catalyzed by ionic liquids. Arayachukiat et al. (2017) and Yingcharoen et al. (2019) investigated the hydrogen-bonding effect of organic catalysts on the cycloaddition of CO_2_ to epoxides.

In addition, the previous theoretical studies mainly focused on elucidating the mechanisms of reactions of CO_2_ with different oxides catalyzed by catalysts. For instance, Sun calculated the reaction pathways of CO_2_ with the propylene oxide catalyzed by the alkylmethylimidazolium chlorine using the density functional theory (DFT) method (Sun and Zhang, 2007). Whiteoak et al. (2012) performed a DFT calculation on the mechanism of CO_2_ conversion to carbonates catalyzed by organic catalyst. Girard et al. (2014) calculated the mechanism of CO_2_ with the styrene oxide catalyzed by BmimBr. Luo et al. (2016) and Huang et al. (2017) performed detailed DFT calculations on the reaction mechanisms of amines, CO_2_ and EO catalyzed by the binary ionic liquids BmimBr and BmimOAc. Further, Ji et al. (2018) researched an efficient catalyst that was composed of imidazolium ionic liquids and organic bases for catalyzing the cycloaddition of CO_2_ and epoxides under atmospheric pressure. The mixed system of ionic liquid and organic base exhibits distinctly higher catalytic activity than using single component alone. Their DFT calculation study on the mechanism of synergistic catalysis revealed that the ionic liquids played an important role in the ring-opening reaction of epoxide and the organic bases facilitated the generation of bicarbonate ion.

In this work, we report a new AFIL catalyst whose design is inspired by the idea of combination of ionic liquids and organic bases. When the new catalyst was used to catalyze the cycloaddition reaction of CO_2_ with epichlorohydrin, it exhibited the synergistic catalysis effect better than the single catalyst of ionic liquid or organic base. The cycloaddition reaction of CO_2_ with epichlorohydrin catalyzed by AFIL catalyst has a good yield of the product of cyclic carbonate. We also performed a detailed DFT calculation on its mechanism to account for the synergistic catalysis effect of AFIL.

## Computational and Experimental Details

### Computational Methods

All the DFT calculations throughout this work were carried out in the Gaussian09 software package (Frisch et al., 2009). The geometrical structures of all reactants, intermediates and products were directly optimized in solution phase. In order to obtain accurate calculated results, we employ the method of functional ωB97X-D (Chai and Head-Gordon, 2008) combined with a large basis set 6-31+G^*^ (Rassolov et al., 2001), denoted as ωB97X-D/6-31+G^*^. The solvent effect is described by the SMD (Dolg et al., 1987; Andrae et al., 1990) model with the value of dielectric constant being 20.8. The frequency analysis is performed at the same computational level to identify the minima or transition states on potential energy surfaces. The calculation conditions for the reaction temperature and pressure were chosen as 353 K and 1 standard atmosphere pressure, which was as same to the experimental conditions. The high temperature was considered to make a significant contribution of *T*Δ*S* in Gibbs free energy and the units for energies are in kcal/mol. More details about the optimized structures are provided in Supplementary Material.

### Experimental Methods

#### Chemicals and Materials

1-Butylimidazole, N,N-diethylbenzylamine (DEBA) and 1,3-bis(bromomethyl)benzene were purchased from J&K with AR grade. CO_2_ was supplied by Doumaoai Factory with a purity of 99.9%. Epichlorohydrin, diethylamine (DEA), potassium carbonate (K_2_CO_3_), acetonitrile, toluene, ether were obtained from Sinopharm with AR grade. All chemicals were used without further purification.

NMR spectra were recorded on Bruker Ascend 400 instruments with tetramethylsilane as the internal standard. GC analysis was performed by using a Shimadzu GC−14 B equipped with a capillary column DM-1701 (60 m-0.32 mm-0.25 mm) equipped with a flame ionization detector.

#### Synthesis of Ionic Liquid Catalysts

1-Butylimidazole (0.188 g, 2 mmol) in acetonitrile (50 mL) and 1,3-bis(bromomethyl)benzene (2.64 g, 10 mmol) in toluene (50 mL) were mixed in a 250 mL round bottom flask and stirred at 85°C for 24 h. The precipitate was filtered and washed with Et_2_O (10 mL × 3) to ensure 1,3-bis(bromomethyl)benzene was removed completely. A white solid (**1**) was obtained after vacuum dried in the yield of 82%. ^1^H NMR (400 MHz, DMSO-d_6_, TMS): δ 9.40 (s, 1H), 7.85 (s, 2H), 7.49–7.35 (m, 4H), 5.46 (s, 2H), 4.70 (s, 2H), 4.20 (t, *J* = 6.0 Hz, 2H), 1.82–1.75 (m, 2H), 1.28–1.23 (m, 2H), 0.90 (t, *J* = 6.0 Hz, 2H).

In a solution of **1** (0.776 g, 2 mmol), K_2_CO_3_ (1.38 g, 10 mmol) and acetonitrile (100 mL), DEA (1.46 g, 20 mmol) was added dropwise. The mixture was stirred at room temperature for 24 h. The oily precipitate was washed with Et_2_O (10 mL × 3). AFIL was obtained as a yellow viscous liquid in the yield of 88% after vacuum dried. ^1^H NMR (400 MHz, DMSO-d6, TMS): δ 9.37 (s, 1H), 7.85 (s, 2H), 7.37–7.26 (m, 4H), 5.43 (s, 2H), 4.20 (t, *J* = 6.0 Hz, 2H), 3.51 (s, 2H), 2.46–2.40 (m, 4H), 1.81–1.74 (m, 2H), 1.27–1.22 (m, 2H), 0.97–0.88 (m, 9H). The ionic liquid 1-benzyl-3-butylimidazolium bromide (BnBimBr) was synthesized by a reported method (Ji et al., 2018).

### Cycloaddition Reaction of CO_2_ and Epichlorohydrin

Epichlorohydrin (0.925 g, 10 mmol) and AFIL (0.38 g, 0.1 mmol) were put into a 50 mL stainless steel autoclave equipped with a magnetic stirrer. The reaction was carried out under atmospheric pressure of CO_2_ at 80°C. After the completion of reaction, the autoclave was cooled to room temperature. In order to remove the ionic liquid, Et_2_O (50 mL) was added to the reaction mixture and filtered. The filtrate was subsequently analyzed by gas chromatography to determine yield using dodecane as internal standard. The product was purified by chromatography on silica gel and characterized structurally by NMR spectroscopy.

3-Chloro-1,2-propylene carbonate: Colorless liquid, ^1^H NMR (400 MHz, CDCl_3_, TMS) δ 5.01–4.95(m, 1H), 4.57 (t, *J* = 8.0 Hz, 1H), 4.39–4.35 (m, 1H), 3.81–3.77 (m, 1H), 3.72–3.68 (m, 1H). ^13^C NMR (100 MHz, CDCl_3_, TMS) δ 154.24, 74.33, 66.99, 43.74.

## Results and Discussion

### Structures of the Ionic Liquid Catalysts

**Figure 1:**
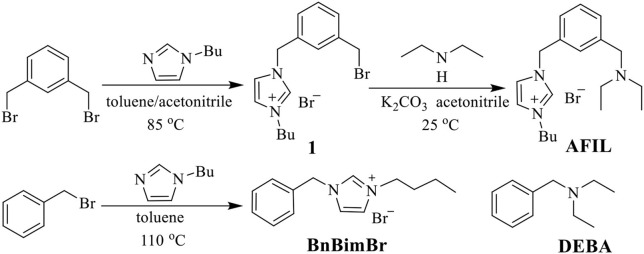
The structures of catalysts for the cycloaddition of CO_2_ and epichlorohydrin.

The structures and synthetic procedures of ionic liquid catalysts for the cycloaddition of CO_2_ and epichlorohydrin were shown in Figure 1.

### Ionic Liquids Catalyzed CO_2_ Cycloaddition Reaction

To investigate the intramolecular synergistic catalytic system of imidazolium and amino functional group in AFIL, BnBimBr, DEBA and AFIL were applied to catalyze the cycloaddition of CO_2_ and epichlorohydrin. The synergistic catalytic effect of organic base and ionic liquid for the conversion of CO_2_ was shown in Table 1. When BnBimBr was used alone, a moderate yield of 50% of 3-chloro-1,2-propylenecarbonate was obtained after 3 h under atmospheric CO_2_ pressure (entry 1, Table 1). If DEBA was used alone, a poor yield of 14% was obtained (entry 2, Table 1). However, the AFIL gave excellent yield of 85% (entry 3, Table 1), indicating the intramolecular synergistic catalytic system of AFIL for accelerating the cycloaddition reaction.

**Table 1:** The synergistic catalytic effect of organic base and ionic liquid for the cycloaddition of CO_2_ and epichlorohydrin.

**Entry**	**Catalyst**	**Yield [%]**
1	BnBimBr	50
2	DEBA	14
3	AFIL	85

Reaction conditions: Epichlorohydrin (10 mmol), CO_2_ (0.1 MPa), catalyst (0.1 mmol), 80°C, 3 h.

**Figure 2:**
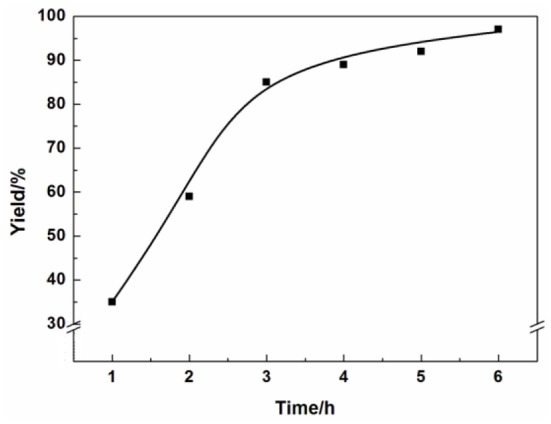
Effect of reaction time.

The optimized experimental conditions such as reaction time, reaction temperature and catalyst amount on the reaction have been systemically investigated. The effect of different reaction times on the product yield was shown in Figure 2. The yield of the 3-chloro-1,2-propylenecarbonate increased with raising reaction time from 1 to 6 h. After 3 h, the growth trend of the yield slowed down obviously. When the reaction time was 6 h, the yield could reach 97%.

Reaction condition: Epichlorohydrin (10 mmol), CO_2_ (0.1 MPa), AFIL (0.1 mmol), 80°C, GC yield.

**Figure 3:**
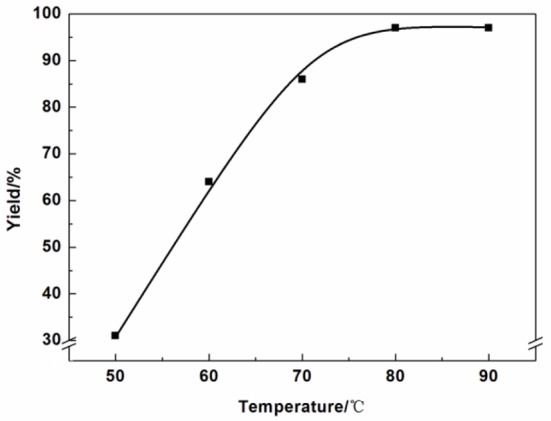
Effect of reaction temperature.

The effect of reaction temperatures was listed in Figure 3. The yield increased significantly with the temperature from 50 to 80°C. When the reaction temperature higher than 80°C, the yield barely increased.

**Figure 4:**
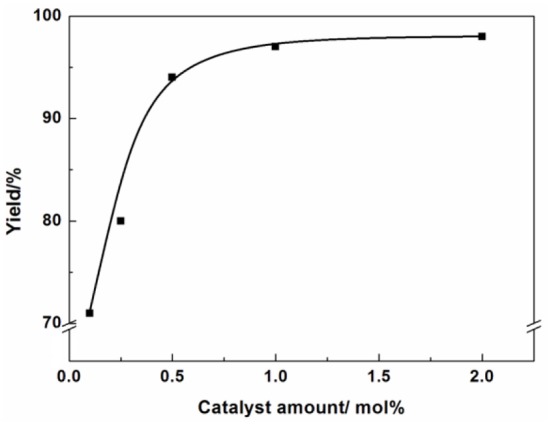
Effect of catalyst amount.

Reaction condition: Epichlorohydrin (10 mmol), CO_2_ (0.1 MPa), AFIL (0.1 mmol), 6 h, GC yield. The effect of catalyst amount on the reaction was also investigated (Figure 4). In the absence of any catalyst, the reaction did not proceed. However, in the presence of the catalyst, the reaction took place immediately. When the catalyst amount increased from 0.1 to 0.5 mol%, the yield increased from 71 to 94%. The highest yield of 3-chloro-1,2-propylenecarbonate was obtained under the amount of AFIL increased to 1 mol%. Further increasing the amount of AFIL to 2 mol%, the yield was not increasing anymore.

Reaction condition: Epichlorohydrin (10 mmol), CO_2_ (0.1 MPa), 80°C, 6 h, GC yield.

### Protonation State of Ionic Liquid AFIL

The synthesized AFIL possesses the dual functions of both ionic liquids and organic bases. The previous experimental and theoretical studies (Elageed et al., 2016; Ji et al., 2018) have revealed that the ionic liquids having imidazolium rings could assist the reaction of ring-opening of epoxides by forming hydrogen bonding interaction with epoxides. On the other hand, it has been reported that the yields of products for CO_2_ conversion catalyzed by the tertiary amines such as DBU (Heldebrant et al., 2005) and DABCO (Ji et al., 2018) increased remarkably in the presence of water. In the reactions of CO_2_ and epoxides catalyzed by ionic liquids, the appropriate amount of water would facilitate the formation of bicarbonate salts effectively. The reason may be that CO_2_ can dissolve in H_2_O to form carbonic acid (H_2_CO_3_) which dissociates as bicarbonate (${\text{HCO}}_{3}^{-}$) and proton. The tertiary amine groups in ionic liquids possess strong basicity so that they can prompt the dissociation of H_2_CO_3_ and increase the dissolution of CO_2_ in solution. In the first step, we should consider the protonation state of amine group of AFIL in solution because the chemical equilibrium between AFIL and H_2_CO_3_ might exist in the solution phase.

**Figure 5:**
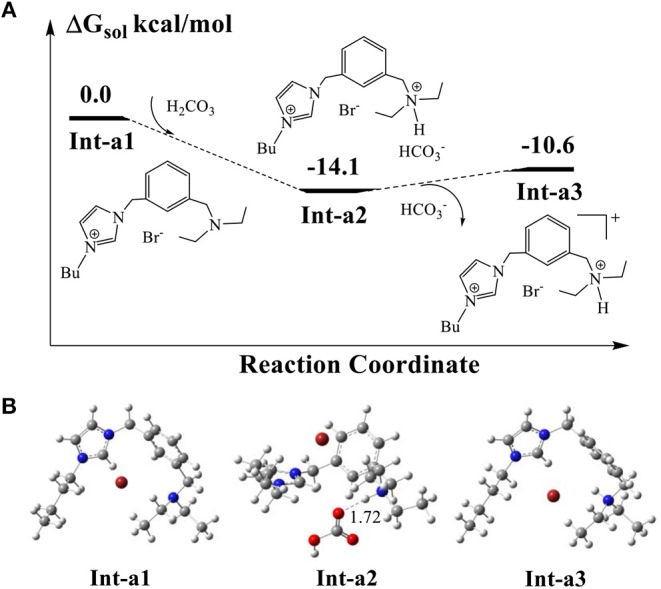
**(A)** Calculated energy profile for proton transfer from H_2_CO_3_ to AFIL. **(B)** Optimized structures of intermediates corresponding to **(A)**. The units of bond lengths are in Å.

The calculated DFT results in Figure 5 indicate that the proton abstraction from H_2_CO_3_ by AFIL, denoted as **Int-a1**, occurs easily to form the complex **Int2-a2**, releasing an energy of 14.1 kcal/mol. The process of proton transfer from carbonic acid to **Int-a1** has no barrier and the formed complex **Int2-a2** is quite stable, which demonstrates that the ionic liquid AFIL exists in a protonated state in solution. The releasing of ${\text{HCO}}_{3}^{-}$ from the complex **Int2-a2** needs only 3.5 kcal/mol and the protonated **Int-a3** is ready for the subsequent catalytic reaction.

### Ring-Opening Reaction of Epichlorohydrin

**Figure 6:**
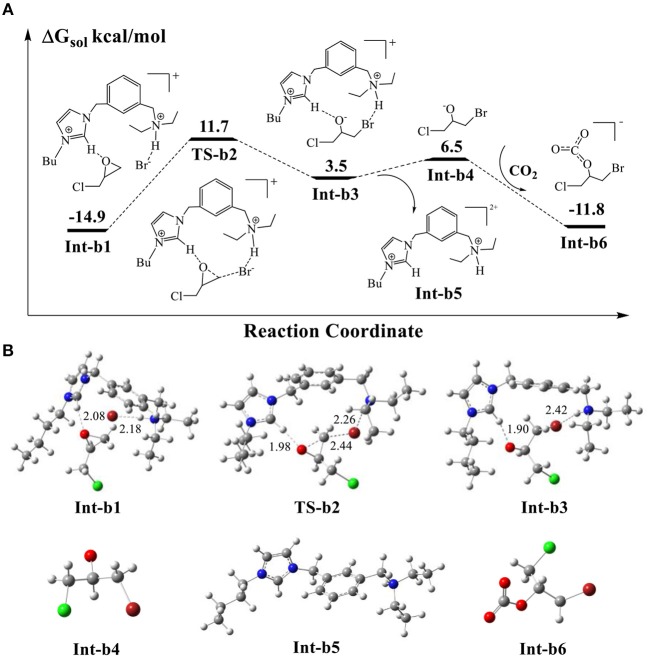
**(A)** Calculated energy profile for the ring-opening reaction of epichlorohydrin assisted by the hydrogen-bond of AFIL. **(B)** Optimized structures of intermediates and transition states corresponding to **(A)**. The units of bond lengths are in Å.

The previous studies (Elageed et al., 2016; Luo et al., 2016; Huang et al., 2017; Ji et al., 2018) supported that the imidazolium rings of ionic liquids could form hydrogen bonding interaction with epoxides. In AFIL, the moiety of 2-buthylimidazole can assist the ring-opening process of epoxides through hydrogen-bonding interaction with them. Figures 6A,B show the calculated energy profile for the ring-opening reaction of epichlorohydrin as well as the corresponding optimized structures. The protonated **Int-a3** in Figure 5 and epichlorohydrin form the stable complex **Int-b1** through the bridged oxygen atom of epichlorohydrin. The calculated energy of **Int-b1** is −14.9 kcal/mol, which is stable than the isolated **Int-a3** and epichlorohydrin by 4.3 kcal/mol. The distance of hydrogen bonding interaction in **Int-b1** is 2.08 Å. In addition, we notice that the Br^−^ stays 2.18 Å away from protonated amine group and forms a weak hydrogen bonding interaction, with the distance being 2.18 Å.

Next, the Br^−^ attacks the carbon atom of epichlorohydrin via the transition state **TS-b2**, with a modest energy barrier of 26.6 kcal/mol. If a count ion ${\text{HCO}}_{3}^{-}$ is considered in this step, the calculated barrier would be 27.7 kcal/mol and the influence of count ion on barrier is not obvious. With the help of hydrogen bonding interaction of imidazole ring, the O-C bond cleaves to give rise to the ring-opening intermediate **Int-b3**. We note in **Int-b3** that the Br^−^ stabilized by amine group forms covalent bond with the carbon atom of cleaved O-C bond. The oxygen atom carries negative charge, which is stabilized by the acid C-H bond of imidazolium ring. Such an oxy anion species is very active so that the addition reaction could occur by the electrophilic attack of free CO_2_. It is noticed that the optimized structure of **Int-b3** in Figure 6 shows that the ring-opening product is embedded in the catalytic cavity formed by the surrounded ionic liquid molecule. The direct addition of CO_2_ to the oxy anion species in the complex **Int-b3** would encounter a high energy barrier due to the steric effect of surrounded molecule. By taking the thermal dynamics of complexes in solution into account, we propose a reaction pathway for the CO_2_ addition. It is possible that the complex **Int-b3** could dissociate into the isolated oxy anion species **Int-b4** and the ionic liquid **Int-b5** by breaking the H-bond interaction, with absorbing an energy of 3.0 kcal/mol. Then, the direct electrophilic attack of CO_2_ to **Int-b4** yields the stable addition product **Int-b6**. This process of CO_2_ addition is extremely exothermic by 18.3 kcal/mol. The DFT scan calculation (Supplementary Material) shows that the energy profile of CO_2_ attack is downhill in energy, without an obvious barrier. For the whole ring-opening process, the O-C bond cleavage in the epichlorohydrin of **Int-b1** is the rate-determining step, with the calculated barrier of 26.6 kcal/mol.

### Generation of Cyclic Carbonate

**Figure 7:**
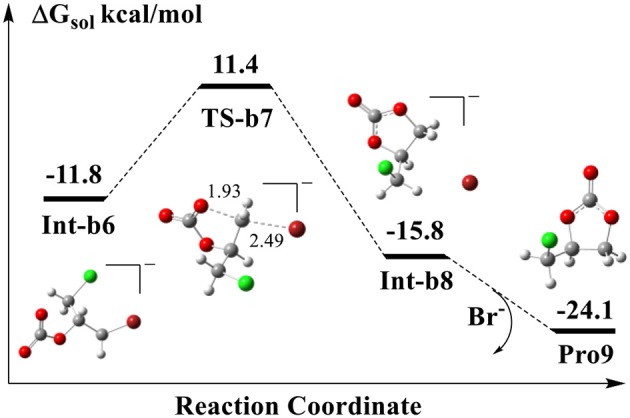
Calculated energy profile for the ring-closing reaction to yield the cyclic carbonate. The units of bond lengths are in Å.

The final step is the intramolecular ring-closing initiated from the substrate **Int-b6**. Figure 7 shows the calculated energy profile for the ring-closing process. As shown in **TS-b7**, one of the oxygen atoms in the carboxyl COO group attacks the terminal carbon atom to form a new O-C bond, while the C-Br bond is being broken at the same time. In the optimized **TS-b7**, the distances of O-C and C-Br atoms are 1.93 and 2.49 Å, respectively, which indicates that it follows a typical S_N2_ mechanism. The five-member-ring complex **Int-b8** is 4.0 kcal/mol lower than that of **Int-b6** in energy. In the structure of **Int-b8**, the Br^−^ has to stay far away from the cyclic carbonate, since the carbonate itself contains a few atoms such as the oxygen atoms that carry negative charges.

We notice that in the structure of **Int-b1** of Figure 6, the Br^−^ anion is stabilized by the N-H bond of amine group. Later, it assists the ring-opening in **TS-b2** by attacking the carbon atom and forms the C-Br bond with the carbon atom in **Int-b3**. After the ring-closing via **TS-b7**, it is evident that the Br^−^ is released again through the cleavage of the covalent C-Br bond. Our DFT calculation shows that if the free Br^−^ reforms the complex **Int-a3** with the **Int-b3** and yields the final cylic carbonate **Pro9**, this process is exothermic in thermodynamics and the energy is lower than that of **Int-b8** by 8.3 kcal/mol. The barrier of rate-determining step for the ring-closing process is 23.2 kcal/mol, lower than that step of ring-opening by 3.4 kcal/mol.

**Scheme 1:**
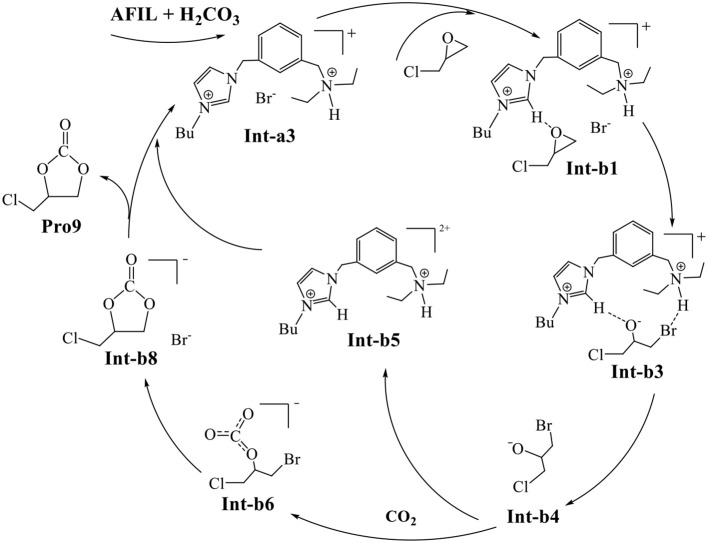
The cycle of CO_2_ conversion catalyzed by AFIL to yield cyclic carbonate.

Finally, the whole mechanism of CO_2_ conversion catalyzed by AFIL is summarized in Scheme 1. The protonated ionic liquid **Int-a3** catalyzes the ring-opening of **Int-b1** to the ring-opening intermediate **Int-b3**. The direct addition of CO_2_ to the released **Int-b4** yields **Int-b6**. The species **Int-b6** undergoes the ring-closing to yield **Int-b8**. The **Int-b8** gives rise to the final product **Pro9** by releasing the Br^−^ who reforms the catalytic **Int-a3** with **Int-b5**.

## Conclusion

In this work, we report a new synthesized amine-functional ionic liquid (AFIL**)** which could catalyze the reaction of CO_2_ and epichlorohydrin to yield cyclic carbonate. The catalytic efficiency of AFIL for converting CO_2_ into cyclic carbonate is much higher than that of the respective BnBimBr and DEBA, which exhibit a bifunctional catalytic activity for CO_2_ conversion. Our DFT calculations indicate that the amine group of AFIL is in a protonated state in solution. The protonated amine group in AFIL could stabilize Br^−^ to attack the carbon atom of epichlorohydrin and assist its ring-opening. Meanwhile, the imidazolium ring in AFIL also could assist the ring-opening of epichlorohydrin through a hydrogen bonding interaction. The rate-determining step of the whole reaction is the ring-opening process, which is in line with the findings in the previous studies. The mechanistic study on the reaction catalyzed by AFIL inspires us to design new multi-functional green catalysts for CO_2_ conversion with high efficiency.

## Data Availability

All datasets generated for this study are included in the manuscript/Supplementary Files.

## Author Contributions

FX and GG designed the experiments, DFT calculations, and analyzed the data. TD and LJ performed experiments. HG performed calculations. All the authors wrote the paper together.

### Conflict of Interest Statement

The authors declare that the research was conducted in the absence of any commercial or financial relationships that could be construed as a potential conflict of interest.
