# Supplementary material for: Mechanistic Studies of CO2 Cycloaddition Reaction Catalyzed by Amine-Functionalized Ionic Liquids
Source: Front Chem. 2019 Sep 10;7:615. doi: 10.3389/fchem.2019.00615 (PMC6747045; doi:10.3389/fchem.2019.00615)
Supplement: Supplementary file 1 [file Table_1.DOCX]

**Supplementary Material**

Energy scan calculation of electrophilic attack of CO_2_ to **Int-b4**


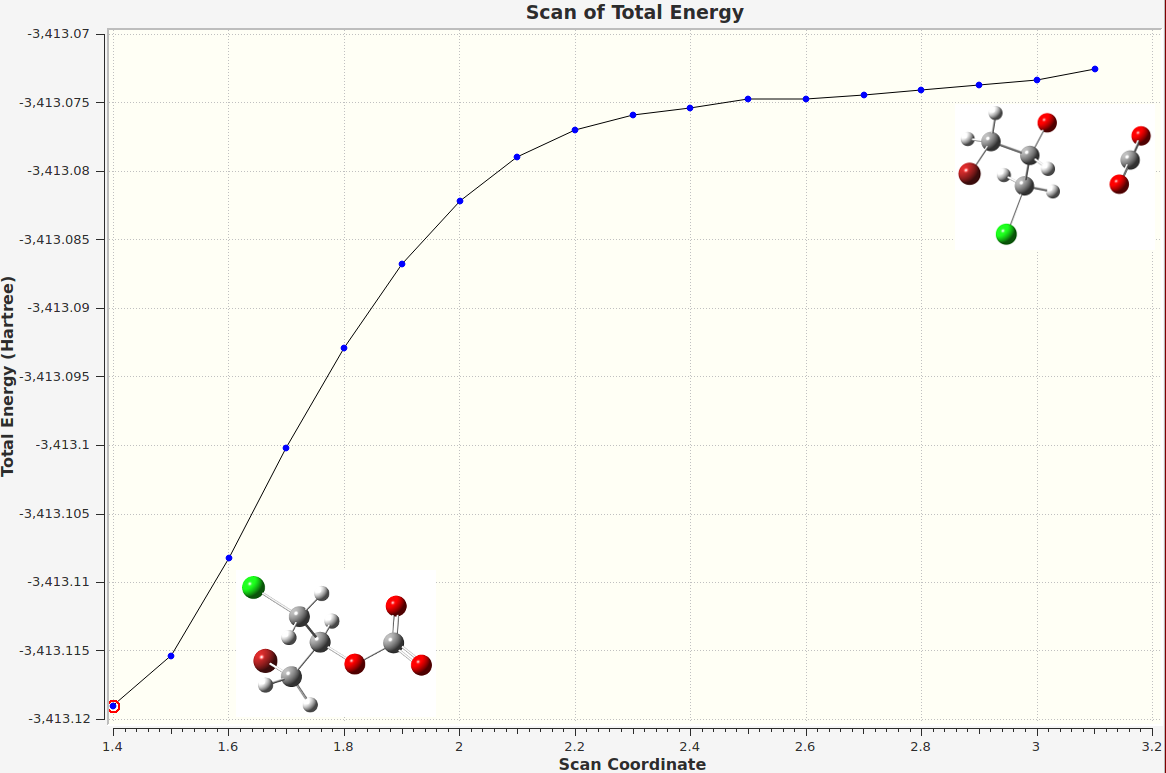


Optimized Int-b1 with HCO_3_^-^ ( ΔG = -4395.058567 a.u.)

**
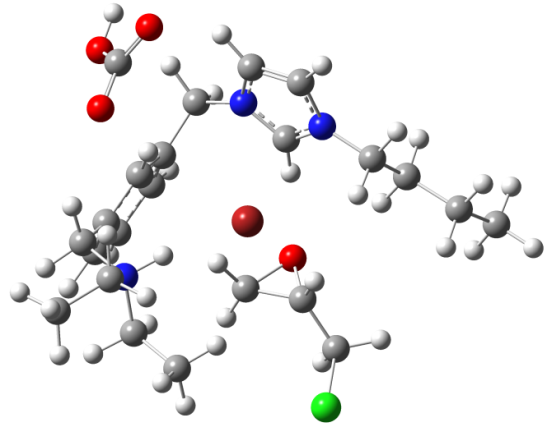
**

Optimized TS-b2 with HCO_3_^-^ ( ΔG = -4395.014326 a.u.)

**
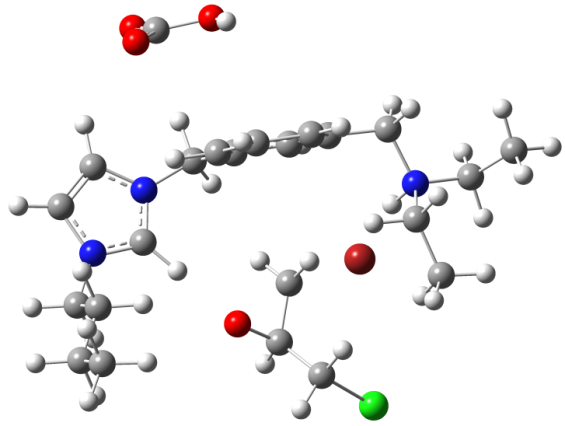
**

**Optimized coordinates of structures in Figure 5-7**

**Int-a1**

C -1.07036100 -2.53424200 -0.34742100

C -1.94881700 -1.51182200 -0.69705900

C -2.88249600 -1.01574800 0.21569200

C -2.94170400 -1.59311500 1.48424500

C -2.08489700 -2.63635100 1.83549300

C -1.14129200 -3.10097200 0.92683700

H -1.89530300 -1.08410200 -1.69550500

H -3.66100600 -1.22113200 2.21168700

H -2.14479000 -3.07567200 2.82828000

H -0.45663300 -3.89926900 1.20598800

C -0.02856500 -2.99517100 -1.33458100

H -0.36878700 -2.84930400 -2.36309200

H 0.20593800 -4.05301100 -1.20348200

C -3.83556500 0.09669300 -0.17192100

H -4.57234700 -0.29886800 -0.88358900

H -4.39994400 0.39623900 0.72908100

C 1.42305000 -0.96680500 -1.38624400

N -3.17726300 1.23685300 -0.80939100

C -4.14988700 2.12017200 -1.46262200

C -5.12122300 2.88104900 -0.55561000

H -4.72406000 1.50252600 -2.16426800

H -3.59580700 2.83898200 -2.07636900

H -5.79779700 3.48304300 -1.17430900

H -5.73718400 2.20498500 0.04878500

H -4.59993000 3.56500400 0.12381700

C -2.29848400 1.92317900 0.14320000

C -1.48690800 3.05225700 -0.47548600

H -1.59506000 1.18696600 0.54620000

H -2.87344300 2.31214700 1.00540200

H -0.75780900 3.41460200 0.25794300

H -0.93061600 2.69908000 -1.35311900

H -2.10262700 3.90687200 -0.78060300

C 2.45753400 -2.82568800 -0.82802100

H 0.65333600 -0.26373600 -1.66253100

H 2.56096000 -3.87717100 -0.60582000

C 3.37603100 -1.82432800 -0.85031400

N 1.24671200 -2.27294700 -1.18758800

N 2.71037600 -0.67606000 -1.22379600

H 4.43634300 -1.83362300 -0.64852300

C 3.29934100 0.65810200 -1.37908800

C 3.95160100 1.16591800 -0.09915800

H 2.48513600 1.31996800 -1.68524600

H 4.02092600 0.60430300 -2.20057100

C 4.50010100 2.58048600 -0.28654400

H 3.20073300 1.14399200 0.69937600

H 4.76970200 0.49314200 0.19144600

C 5.14571400 3.12054800 0.98763100

H 5.23606400 2.58861300 -1.10262300

H 3.68359600 3.24895900 -0.59295400

H 5.54339600 4.13098100 0.83493700

H 4.41817800 3.16653300 1.80739600

H 5.97600000 2.48063000 1.31238100

Br 0.75957200 0.23395500 1.61774700

**Int-a2**

C 2.99204500 0.43352000 -0.06494500

C 3.83772300 -0.60051200 -0.46372400

C 4.86679500 -1.04557400 0.36643100

C 5.08405400 -0.39902100 1.58400600

C 4.26952100 0.66211100 1.97107200

C 3.21503900 1.06639600 1.15834000

H 3.68853900 -1.07537400 -1.43178000

H 5.88977100 -0.73034800 2.23533000

H 4.44441700 1.16163900 2.92028200

H 2.56311800 1.87707200 1.47536500

C 1.83340900 0.83743500 -0.93939500

H 2.11070400 0.81634700 -1.99724500

H 1.49067900 1.84447400 -0.69642200

C 5.72387900 -2.21039000 -0.05617200

H 6.00122300 -2.12282400 -1.10989400

H 6.63550700 -2.25825800 0.54291000

C 0.70268200 -1.37693800 -0.66291000

N 5.01832600 -3.52961000 0.08141000

C 5.79034400 -4.63812200 -0.57292400

C 7.08065900 -5.01086200 0.13243800

H 5.98119800 -4.30834500 -1.59715500

H 5.11266800 -5.49229800 -0.62977400

H 7.54480500 -5.83180700 -0.42406200

H 7.79696600 -4.18383100 0.15678100

H 6.91002100 -5.36066500 1.15514800

C 4.61002700 -3.80489100 1.49804500

C 3.89857800 -5.13535700 1.66100800

H 3.92296300 -3.00258600 1.78049800

H 5.50841300 -3.74193500 2.11838200

H 3.49320200 -5.17778900 2.67658400

H 3.05611500 -5.22099600 0.96612800

H 4.56321000 -5.99482700 1.52635500

C -0.65515200 0.35180900 -0.81330200

H 1.58388900 -1.99846900 -0.60265900

H -0.93197400 1.39190700 -0.89870900

C -1.41403800 -0.77249800 -0.73979200

N 0.66657300 -0.04877400 -0.76985400

N -0.54330900 -1.84024600 -0.66360500

H -2.48540300 -0.90447200 -0.74582300

C -0.90178200 -3.25719100 -0.55144100

C -1.65649000 -3.56481000 0.73566200

H 0.03743300 -3.81372300 -0.58977000

H -1.49035400 -3.51676500 -1.43721100

C -1.97989300 -5.05493900 0.84272100

H -1.03181800 -3.25051500 1.58063000

H -2.58818700 -2.98370000 0.76718000

C -2.76629000 -5.38575600 2.10923800

H -2.55416600 -5.37382300 -0.03844800

H -1.04388400 -5.63009100 0.83180100

H -2.98378200 -6.45863200 2.17261400

H -2.20252700 -5.10523100 3.00803200

H -3.72246800 -4.84769000 2.13300700

C 2.79200700 -3.40249200 -2.58221400

O 3.83157700 -3.10877100 -3.21075800

O 2.67798200 -3.70871700 -1.36873900

O 1.59425100 -3.38377200 -3.26336800

Br 1.46835600 -2.12476900 2.42883700

H 4.12950100 -3.47777300 -0.47411600

H 1.77010100 -3.14036900 -4.18612200

**Int-a3**

C 0.92260100 2.61219600 -0.19727800

C 1.86592300 1.70415500 -0.67020900

C 2.82185000 1.15429100 0.18731300

C 2.84818100 1.55154500 1.52280700

C 1.92857700 2.48657600 1.99461200

C 0.96099300 3.00526600 1.14201500

H 1.85373500 1.41659900 -1.72177800

H 3.58747300 1.12828200 2.19902000

H 1.95684500 2.79466100 3.03624100

H 0.23049600 3.71889600 1.51653100

C -0.14459800 3.14099600 -1.12136800

H 0.18854700 3.12159900 -2.16202400

H -0.41122900 4.16943600 -0.87117300

C 3.81163800 0.14893300 -0.33972200

H 4.34000300 0.53823100 -1.21357000

H 4.54439300 -0.12376400 0.42098900

C -1.51778600 1.06471500 -1.31403500

N 3.13559500 -1.12265400 -0.78692800

C 4.03230300 -1.96511200 -1.65587000

C 5.19697200 -2.58703800 -0.91229000

H 4.37789600 -1.30634700 -2.45586900

H 3.39616900 -2.72977100 -2.10451000

H 5.78821800 -3.15891400 -1.63465200

H 5.85620600 -1.83564800 -0.46701900

H 4.86703200 -3.28029100 -0.13302500

C 2.52060100 -1.85680000 0.37328100

C 1.76781300 -3.09971200 -0.05589800

H 1.82088300 -1.15769600 0.83976200

H 3.32796700 -2.08128100 1.07380400

H 1.22323700 -3.47728300 0.81445200

H 1.02658000 -2.86493300 -0.82816300

H 2.42656700 -3.89605400 -0.41665600

C -2.63215200 2.85634400 -0.68831200

H -0.71783500 0.40619800 -1.61075700

H -2.77825900 3.89131700 -0.41790200

C -3.51245800 1.82610600 -0.78454700

N -1.39236000 2.36313700 -1.03841400

N -2.79645900 0.72067100 -1.19487200

H -4.57678100 1.78823600 -0.60871200

C -3.33447100 -0.62855800 -1.40144900

C -3.95004000 -1.20613400 -0.13296600

H -2.49970600 -1.24645400 -1.74234500

H -4.06830600 -0.56786000 -2.21124100

C -4.47231000 -2.62318700 -0.36890300

H -3.18291400 -1.20483600 0.65034100

H -4.77508400 -0.56339300 0.20169900

C -5.10417500 -3.21923800 0.88697000

H -5.21119600 -2.61584600 -1.18225000

H -3.64508700 -3.26550600 -0.70134500

H -5.47485100 -4.23440000 0.70189200

H -4.37633500 -3.27221300 1.70609900

H -5.95110600 -2.61154100 1.22977900

Br -0.63382000 -0.47302400 1.48117700

H 2.35595200 -0.84371700 -1.39222000

**Int-b1**

C -0.77465300 -0.56664900 -0.06629000

C 0.59294100 -0.44851500 -0.31915800

C 1.05184900 0.06030400 -1.53477400

C 0.12533400 0.42542700 -2.51697000

C -1.23924700 0.31443300 -2.27061900

C -1.68833500 -0.16946800 -1.04248200

H 1.30917600 -0.75458800 0.43960500

H 0.47629700 0.80503700 -3.47378800

H -1.95554500 0.61074100 -3.03171100

H -2.75545800 -0.24767800 -0.84837100

C -1.26681600 -1.15099500 1.23653500

H -0.93265600 -2.18480300 1.35171900

H -2.35890900 -1.14724500 1.27348900

C 2.53224200 0.21532700 -1.77158300

H 3.08371800 -0.58799100 -1.27785600

H 2.76847300 0.19579600 -2.83769100

C -0.99965500 0.85735900 2.67740600

N 3.06484400 1.50780100 -1.21356900

C 4.55227600 1.46259300 -1.01638900

C 5.35338500 1.40455000 -2.30242500

H 4.74324400 0.58709900 -0.39067500

H 4.81342600 2.34481400 -0.42851300

H 6.41412800 1.35369000 -2.03643600

H 5.11769600 0.51745600 -2.89877500

H 5.20931500 2.29600000 -2.92021700

C 2.58475400 2.69212800 -1.99759000

C 3.02835400 4.01908700 -1.41082800

H 1.49596200 2.63021000 -2.00309700

H 2.92914000 2.56531700 -3.02657400

H 2.50306900 4.81847700 -1.94355900

H 2.77148300 4.09209100 -0.34913900

H 4.10207300 4.19189900 -1.53032900

C -0.01374100 -0.96192900 3.43534500

H -1.53824200 1.53905000 2.03000700

H 0.30060300 -1.99465000 3.43474100

C 0.18806800 0.03445900 4.33615400

N -0.76922900 -0.42931700 2.41359500

N -0.45709600 1.15313300 3.85658000

H 0.72617600 0.04405300 5.27180200

C -0.36829900 2.49299100 4.44917900

C -1.64728300 3.30329300 4.29223700

H 0.48420800 2.99130700 3.97620100

H -0.13667700 2.34427700 5.50672600

C -1.48782600 4.69682000 4.90089600

H -1.90395700 3.40645200 3.23184500

H -2.47732400 2.77424700 4.77831500

C -2.74978800 5.54200800 4.74747800

H -1.23575400 4.60371300 5.96589200

H -0.64170600 5.20839200 4.42203100

H -2.61586100 6.53578900 5.19041800

H -3.00831000 5.67950400 3.69001800

H -3.60661600 5.06804500 5.24248700

Br 2.15430700 1.98971400 1.84471300

C -0.74275300 4.13669800 0.82238400

C -0.75406700 3.20282800 -0.30133100

O -1.76564700 3.13789700 0.71912800

H -0.02431500 3.95913000 1.62146100

H -0.05717900 2.36823100 -0.28490900

H -1.11532500 3.53278200 -1.27385600

C -1.26373400 5.53783800 0.67976200

H -1.70314200 5.89775000 1.61158000

H -1.99583100 5.60887600 -0.12657800

Cl 0.09668700 6.66025900 0.27984000

H 2.67784500 1.61528500 -0.24267300

**TS-b2**

C -8.81369300 3.15979200 0.00454600

C -7.71606300 3.24261800 -0.85200800

C -7.88143000 3.57962400 -2.19801800

C -9.16627200 3.82327300 -2.68829600

C -10.26578500 3.74709100 -1.83755700

C -10.09104500 3.42119400 -0.49518400

H -6.71790400 3.04718100 -0.46462300

H -9.30590300 4.08103800 -3.73525800

H -11.26332600 3.94146400 -2.22139900

H -10.95832400 3.36977800 0.15912200

C -8.59594600 2.75164800 1.44615800

H -7.59373400 3.02398900 1.78441900

H -8.69753000 1.66964600 1.56518300

C -6.68433100 3.69107000 -3.10774700

H -5.95770700 2.90357400 -2.89441700

H -6.98491500 3.61533800 -4.15396800

C -9.63141300 4.66605400 2.63564600

N -5.94855500 4.99592300 -2.94830200

C -4.64470300 4.99389600 -3.70047000

C -4.79160000 5.02033500 -5.20869000

H -4.11302200 4.09785500 -3.37101600

H -4.08206400 5.85841200 -3.34328700

H -3.78640800 4.99662500 -5.64154900

H -5.33618300 4.15224600 -5.59143500

H -5.28226300 5.93241500 -5.56122200

C -6.82046300 6.18087300 -3.24188900

C -6.10996500 7.50456800 -3.03015200

H -7.67705900 6.10403100 -2.57013700

H -7.18722300 6.06980600 -4.26512800

H -6.85721000 8.30208300 -3.08808500

H -5.63819600 7.55242700 -2.04298900

H -5.35168100 7.70133700 -3.79362000

C -10.63999400 2.72805800 2.94676400

H -8.95663300 5.44675700 2.28285700

H -10.79848400 1.66471900 2.84674900

C -11.34490600 3.68027300 3.61283400

N -9.56910900 3.36307500 2.35188700

N -10.69214800 4.87778000 3.41000800

H -12.24086900 3.61299400 4.21152500

C -11.13447700 6.20326100 3.85816300

C -11.94809600 6.91255800 2.78195000

H -10.23688800 6.77228900 4.11401100

H -11.71552900 6.05228600 4.77089200

C -12.39988800 8.29744000 3.24388700

H -11.33494100 7.00487100 1.87563900

H -12.82187800 6.29845400 2.52548000

C -13.19234800 9.03579000 2.16799100

H -13.01151200 8.19799100 4.15076100

H -11.51816300 8.89060600 3.52193700

H -13.50863200 10.02506900 2.51907600

H -12.59029200 9.17801100 1.26190600

H -14.09407900 8.47745300 1.88618300

Br -4.61417900 5.35205400 0.03857700

C -6.82446700 7.40373600 1.45463300

C -6.82169700 6.12051900 0.74283700

O -8.18840500 7.13635600 1.58836000

H -6.26164800 7.41441500 2.39688300

H -6.91592400 5.22304000 1.33363200

H -7.21721200 6.11467800 -0.26439900

C -6.60870600 8.67493300 0.65410500

H -7.11550400 9.50151200 1.15595900

H -6.99946800 8.57062200 -0.36027000

Cl -4.86857700 9.15417100 0.51814000

H -5.65943700 5.06611600 -1.95066700

**Int-b3**

C -1.50846800 -1.62644900 -0.03059000

C -0.40500400 -1.53639900 -0.87897200

C -0.56568900 -1.21559200 -2.22916100

C -1.84952500 -0.99868700 -2.73383000

C -2.95434700 -1.08194600 -1.89133100

C -2.78496300 -1.38885100 -0.54395700

H 0.59136900 -1.72569200 -0.48183100

H -1.98372200 -0.75086600 -3.78376300

H -3.95138100 -0.90640600 -2.28527400

H -3.65597900 -1.44327100 0.10493000

C -1.30163400 -2.01790600 1.41651900

H -0.29977600 -1.74865500 1.75768400

H -1.41107700 -3.09797500 1.54612100

C 0.63860200 -1.06869700 -3.12130200

H 1.38977400 -1.83035600 -2.90000600

H 0.36051500 -1.13945900 -4.17349700

C -2.31716900 -0.08549600 2.59555600

N 1.32496200 0.26280500 -2.93276600

C 2.64931600 0.31744400 -3.65202900

C 2.53361300 0.35938800 -5.16258900

H 3.20453700 -0.56367500 -3.32071200

H 3.16992600 1.19972700 -3.27458000

H 3.54863100 0.38553400 -5.57129700

H 2.03481100 -0.52465100 -5.57008800

H 2.01397800 1.25628000 -5.51215100

C 0.41221900 1.42023100 -3.23191400

C 1.06679600 2.76791400 -3.00104700

H -0.45005900 1.30437400 -2.57345400

H 0.07011600 1.29978800 -4.26198200

H 0.29279200 3.53680800 -3.08788000

H 1.49834300 2.84400500 -1.99742000

H 1.84381800 2.99180600 -3.73745100

C -3.36759700 -2.00917500 2.88292600

H -1.62288700 0.70265700 2.26872900

H -3.54311300 -3.06903800 2.77433200

C -4.06449400 -1.04818900 3.54406300

N -2.27620600 -1.38945400 2.30753500

N -3.38607100 0.13786300 3.35730100

H -4.96975900 -1.10258500 4.12999800

C -3.80457700 1.46719200 3.81578100

C -4.60239100 2.20279700 2.74552500

H -2.89636300 2.01687900 4.07591000

H -4.38933200 1.32014700 4.72686600

C -4.98265500 3.61091700 3.20151200

H -3.99788900 2.26087700 1.83056800

H -5.50626000 1.62657000 2.50594500

C -5.77632300 4.36966600 2.14079600

H -5.56927900 3.55016200 4.12835200

H -4.06789000 4.16818800 3.44378200

H -6.02357400 5.38208100 2.48105600

H -5.20362400 4.46019400 1.20921800

H -6.71747800 3.85633900 1.90688500

Br 2.58410500 0.90192200 0.19621300

C 0.43318800 2.55610400 1.50717500

C 0.70036300 1.29671200 0.67456400

O -0.86891400 2.42738300 1.94597800

H 1.17298600 2.60835800 2.32958400

H 0.39462000 0.42072300 1.24130200

H 0.15271000 1.34048700 -0.26996800

C 0.51372000 3.85568400 0.69300200

H 0.03559000 4.65314000 1.26242700

H 0.01180300 3.74519400 -0.27184600

Cl 2.19682200 4.46579000 0.33718100

H 1.57430000 0.32997100 -1.93570100

**Int-b4**

C -0.05472600 -1.95061000 -0.37902400

C -1.42803800 -2.19661000 -0.43579800

C -2.18842900 -2.28871700 0.73041300

C -1.55653300 -2.17320800 1.97203500

C -0.18425800 -1.95448700 2.03499000

C 0.56480100 -1.83575500 0.86511100

H -1.91163800 -2.31680200 -1.40395500

H -2.13833200 -2.25393600 2.88689100

H 0.30821800 -1.86192000 2.99861500

H 1.63260000 -1.64832000 0.94229800

C 0.68959800 -1.77673900 -1.68620700

H 0.46718000 -0.80072600 -2.12740800

H 0.39163300 -2.53911400 -2.40966900

C -3.67372200 -2.51531000 0.63263300

H -3.90544300 -3.23594000 -0.15412700

H -4.08889400 -2.87697800 1.57422400

C 2.94742300 -0.80216900 -1.39058700

N -4.41519600 -1.24596400 0.27805900

C -5.80489300 -1.52994400 -0.23683900

C -6.74878700 -2.08095800 0.81137700

H -5.67381400 -2.23207700 -1.06294700

H -6.17796900 -0.59338600 -0.65507800

H -7.70607300 -2.28310400 0.32062000

H -6.39103300 -3.02072900 1.24214800

H -6.93548000 -1.36626700 1.61835000

C -4.35958600 -0.24527100 1.40103300

C -5.07378600 1.05645200 1.09237900

H -3.30048600 -0.06848300 1.59307200

H -4.77808400 -0.73459600 2.28288600

H -4.83232900 1.76738600 1.88837700

H -4.74878700 1.49514400 0.14266600

H -6.16079900 0.93877900 1.06851200

C 2.87414400 -3.00521500 -1.35826400

H 2.65593300 0.23787700 -1.46372800

H 2.42196100 -3.98360700 -1.42147200

C 4.15631200 -2.62273800 -1.12405700

N 2.13706100 -1.85200200 -1.53257800

N 4.17812700 -1.24280000 -1.15052600

H 5.04885000 -3.20528400 -0.95301500

C 5.34622000 -0.38570600 -0.91269400

C 5.92721400 -0.58506900 0.48170100

H 5.00599200 0.64342100 -1.05102100

H 6.08377500 -0.61290800 -1.68765300

C 7.04480600 0.42184200 0.75533700

H 5.12902100 -0.46365300 1.22404600

H 6.31675700 -1.60694800 0.57787600

C 7.67036300 0.23330100 2.13519900

H 7.82083300 0.32960500 -0.01704000

H 6.63685500 1.43804900 0.67286200

H 8.45405100 0.97775400 2.31863700

H 6.91721000 0.33778400 2.92619800

H 8.12431000 -0.76094800 2.23370800

Br -2.14595100 1.09032000 -1.60494500

C -0.10842000 2.21132500 0.15474800

C -1.40903400 1.40398200 0.17932500

O 0.78988500 1.53870600 1.03112700

H 0.32500600 2.24506000 -0.84735100

H -1.22433700 0.41508500 0.59443300

H -2.19702800 1.89901400 0.74807800

C -0.27101000 3.62758300 0.69737000

H 0.68567800 4.15078500 0.67405900

H -0.66193300 3.61176400 1.71658300

Cl -1.42569000 4.58992500 -0.30344700

H -3.91552800 -0.82621600 -0.51637200

C 2.15761900 1.57970400 0.74667500

O 2.84094100 0.95324500 1.58132000

O 2.52711600 2.19377200 -0.27600400

**Int-b5**

C 0.32271600 -0.58838900 0.10634200

C 1.67227900 -0.85674700 -0.12054400

C 2.48892700 0.07561600 -0.76672900

C 1.93770400 1.27941700 -1.20868500

C 0.58777300 1.54810900 -0.99521700

C -0.21609900 0.62258900 -0.33535400

H 2.09407700 -1.80695900 0.20387600

H 2.56336700 2.00548900 -1.72215500

H 0.15925400 2.48325700 -1.34462700

H -1.26824600 0.84822300 -0.17682700

C -0.51272900 -1.60799400 0.85132000

H -0.52103800 -1.39939700 1.92463100

H -0.11154100 -2.61419500 0.71248900

C 3.94614300 -0.22826800 -0.99433000

H 4.08452500 -1.26326200 -1.31454000

H 4.37383200 0.43557700 -1.74717000

C -2.92815200 -1.06091900 1.05833700

N 4.76574300 -0.05923300 0.26151400

C 6.11505700 -0.72205400 0.14288100

C 7.02527600 -0.09250500 -0.89160200

H 5.90949300 -1.76831900 -0.09377700

H 6.56283200 -0.68952000 1.13692200

H 7.97202400 -0.64187600 -0.87642200

H 6.61659900 -0.16517300 -1.90373100

H 7.24662300 0.95582900 -0.67020300

C 4.81602100 1.37849300 0.70380200

C 5.57820000 1.56834300 2.00076000

H 3.77687200 1.68914400 0.82574600

H 5.25023400 1.95221200 -0.11715000

H 5.41662600 2.59526100 2.34279700

H 5.21144000 0.89304600 2.78216500

H 6.65537500 1.42332900 1.88052100

C -2.35471600 -2.07322900 -0.81402300

H -2.87703800 -0.59912400 2.03371500

H -1.69436700 -2.55663800 -1.51830100

C -3.68284200 -1.79684400 -0.87442700

N -1.90488100 -1.61494300 0.40712900

N -4.02151500 -1.17175700 0.30824300

H -4.41529000 -1.99626800 -1.64209100

C -5.33967100 -0.61448600 0.63377400

C -5.54301100 0.75067900 -0.01341600

H -5.40371100 -0.55379100 1.72287900

H -6.08311300 -1.33687000 0.28766800

C -6.93383900 1.31085200 0.28500700

H -4.77222100 1.43966500 0.35699700

H -5.40383200 0.65914300 -1.09851900

C -7.15906100 2.67697800 -0.35900700

H -7.69339500 0.60483900 -0.07677500

H -7.07064500 1.39018300 1.37186800

H -8.16149500 3.05863600 -0.13363000

H -6.43114900 3.41216900 0.00663400

H -7.05985700 2.62022100 -1.45021500

H 4.27849300 -0.57114900 1.00569600

**Int-b6**

Br 2.17486000 -0.94518300 0.19788700

C -0.54822800 0.05426300 -0.14162800

C 0.58755900 -0.63736000 -0.89937600

O -1.74922200 -0.54028700 -0.63128000

H -0.46977600 -0.11514000 0.93495500

H 0.26305000 -1.62673200 -1.22082300

H 0.91731000 -0.06600800 -1.76871000

C -0.66312900 1.54739500 -0.43887700

H -1.51069400 1.97236100 0.09957900

H -0.78740000 1.71329800 -1.51092300

Cl 0.79560200 2.47873600 0.07472400

C -2.87491400 -0.55115800 0.20170100

O -2.76832100 -0.05068800 1.33913200

O -3.86565300 -1.08574100 -0.34052100

**TS-b7**

O -0.40635800 -1.54838400 0.01939900

C 0.58833100 -1.34989100 1.03549200

H 1.06378000 -2.31861900 1.17501900

C 1.57093200 -0.32260700 0.50894000

H 1.82409000 0.56377600 1.07024200

Br 3.71417100 -1.30255800 1.32132500

H 2.01460000 -0.47041800 -0.46238400

C -0.17831800 -0.92916900 2.28976900

H -0.92717600 -1.68915100 2.51396500

H -0.66923600 0.03467900 2.14407300

Cl 0.85984200 -0.77076600 3.75054100

C -0.69651200 -0.37880300 -0.64482800

O 0.08652600 0.58390500 -0.34893900

O -1.63971800 -0.38805700 -1.43628100

**Int-b8**

O -1.82277100 -0.52919300 1.09891900

C -0.48101100 -0.19387200 0.67357000

H 0.20968300 -0.64534200 1.38636800

C -0.39193400 -0.85764200 -0.70583600

H -0.00028800 -0.21093300 -1.48894700

Br 2.93865100 -0.26936900 -0.01355000

H 0.15451800 -1.79971100 -0.68030100

C -0.28528900 1.30428600 0.73102500

H 0.75183500 1.52876400 0.47476500

H -0.51834500 1.67963400 1.72825500

Cl -1.35165900 2.19511100 -0.42696100

C -2.52554900 -1.01613900 0.07151800

O -1.77276800 -1.15499700 -1.01777400

O -3.70062400 -1.29846000 0.13020400

**Pro9**

O 0.72144900 0.07086200 1.09391800

C -0.18960600 1.06712900 0.59150200

H -0.12719900 1.93006700 1.25749400

C 0.38179200 1.37412700 -0.80030100

H -0.36175500 1.35773700 -1.59750800

H 0.94142100 2.31009500 -0.82082800

C -1.60987400 0.54741100 0.64692700

H -2.29634500 1.31801600 0.29153400

H -1.87113900 0.26671400 1.66795200

Cl -1.85313100 -0.91642000 -0.38185100

C 1.50916000 -0.37653500 0.10791300

O 1.30844400 0.29629300 -1.02651500

O 2.30378300 -1.27669000 0.23466900
